# Supplementary material for: Content-rich biological network constructed by mining PubMed abstracts
Source: BMC Bioinformatics. 2004 Oct 8;5:147. doi: 10.1186/1471-2105-5-147 (PMC528731; doi:10.1186/1471-2105-5-147)
Supplement: Additional File 2 — The original results of the above study (non-essential files are deleted to keep the file size under the limit set by BMC bioinformatics). [file 1471-2105-5-147-S2.bz2 › chilibotAdditionalFile2/dip20/481ID8702798E10571/html/IL8RB_CXCL5.html]

 


 **IL8RB** and **CXCL5** 
  
Found 16 abstracts in PubMed, retrieved 16.  
 

 What does Google say? 
 PDF only 
| .edu only 

---

**Interactive relationship** (e.g. stimulation, inhibition, etc)

**Neutral relationship**- IL 8 activates neutrophils by binding to two distinct seven transmembrane 7 TMR G protein coupled receptors CXCR1 IL 8RA and CXCR2  [ **IL8RB** ]  IL 8RB, while GROalpha, GRObeta, GROgamma, and ENA 78  [ **CXCL5** ]  bind to and activate only CXCR2  [ **IL8RB** ] .  Ref: 9553055 J Biol Chem, 1998
- From these data, it can be concluded that, in addition to IL 8, GCP 2, but not ENA 78  [ **CXCL5** ] , efficiently binds to both CXCR1 and CXCR2  [ **IL8RB** ] .  Ref: 9692902 Eur J Biochem, 1998
- GROalpha, GRObeta, GROgamma, NAP 2, and ENA 78  [ **CXCL5** ]  competed weakly for the high affinity IL 8 binding site at IL8RA  [ **IL8RB** ] .  Ref: 8702798 J Biol Chem, 1996
- The rank order of agonist potency, based on inspection of the mean effective concentration values EC50, for  **IL8RB**  was GROgamma 1 nM > IL 8 4 nM approximately GROalpha 5 nM approximately GRObeta 4 nM approximately NAP 2 7 nM > ENA 78  [ **CXCL5** ]  11 nM, and for IL8RA  [ **IL8RB** ]  was IL 8 4 nM >>> ENA 78  [ **CXCL5** ]  40 nM approximately NAP 2 45 nM > GROalpha 63 nM approximately GROgamma 65 nM >> GRObeta.  Ref: 8702798 J Biol Chem, 1996
- All six agonists for  **IL8RB**  competed for high affinity 125I IL 8, GROalpha, NAP 2, and ENA 78  [ **CXCL5** ]  binding sites at  **IL8RB** .  Ref: 8702798 J Biol Chem, 1996
- CXCR2  [ **IL8RB** ]  binds IL 8 and other structurally related chemokines such as growth related oncogene GRO a, GRObeta, GROgamma, neutrophil activating peptide 2 NAP 2 and epithelial cell derived neutrophil activating peptide 78 ENA 78  [ **CXCL5** ] .  Ref: 12365735 Fish Shellfish Immunol, 2002
- GCP 2 desensitized the calcium increase induced by IL 8 in both CXCR1 transfected and CXCR2  [ **IL8RB** ]  transfected cells, but ENA 78  [ **CXCL5** ]  only affected the IL 8 induced calcium response in CXCR2  [ **IL8RB** ]  transfectants.  Ref: 9692902 Eur J Biochem, 1998

**Non-interactive relationship** (e.g. studied together, co-existance, homology, etc.)

- Thus in COPD, in addition to CXCL8 and CXCR1,  **CXCL5**  and CXCR2  [ **IL8RB** ]  appear to play important roles in the airway neutrophilia characteristic of severe exacerbations.  Ref: 12857718 Am J Respir Crit Care MedAm J Respir Crit Care Med, 1998
- The half maximal effective concentrations for migration of CXCR2  [ **IL8RB** ]  transfectants in response to GCP 2 and ENA 78  [ **CXCL5** ]  were similar 0.1 nM.  Ref: 9692902 Eur J Biochem, 1998
- ... for ENA 78  [ **CXCL5** ] , threefold higher concentrations were necessary to obtain a calcium response in CXCR1 transfected cells than in CXCR2  [ **IL8RB** ]  transfectants.  Ref: 9692902 Eur J Biochem, 1998
- By contrast, the migratory response to the CXCR2  [ **IL8RB** ]  ligands, epithelial cell derived neutrophil activator ENA 78  [ **CXCL5** ]  and the growth related oncogene proteins, was markedly suppressed in PMN from septic patients p <.  Ref: 9973513 J Immunol, 1999
- By measuring calcium flux in human embryonic kidney 293 cells transfected with plasmids encoding IL8RA  [ **IL8RB** ]  or  **IL8RB** , we have now defined receptor selectivity for GRObeta, GROgamma, and ENA 78  [ **CXCL5** ] .  Ref: 8702798 J Biol Chem, 1996
- Radiolabeled IL 8 could efficiently be displaced from CXCR2  [ **IL8RB** ]  by IL 8, GCP 2 and ENA 78  [ **CXCL5** ] .  Ref: 9692902 Eur J Biochem, 1998
- RESULTS Flow cytometry and radioligand binding data indicate that IL 8, GCP 2, and ENA 78  [ **CXCL5** ]  equivalently reduced CXCR 1 and CXCR 2  [ **IL8RB** ]  cell surface expression by 34% to 54%.  Ref: 10593335 Arch Surg, 1999
- Using Jurkat cells that stably express either CXCR1 or CXCR2  [ **IL8RB** ] , we studied receptor activation by IL 8, GCP 2 epithelial neutrophil activating protein 2 ENA 78  [ **CXCL5** ]  which shares 77% identical amino acids with GCP 2 and growth regulated oncogene alpha GRO alpha.  Ref: 9485196 Eur J Immunol, 1998
- By comparison with the NSC and S COPD groups, neutrophilia and gene expression for epithelial derived neutrophil attractant 78  **CXCL5** , interleukin 8 CXCL8, CXCR1 and CXCR2  [ **IL8RB** ]  were each up regulated in the E COPD group p&lt.  Ref: 12857718 Am J Respir Crit Care MedAm J Respir Crit Care Med, 1998
- CONCLUSION These findings suggest that, through expression of  **CXCL5** , eosinophils can recruit and activate CXC receptor 2 CXCR2  [ **IL8RB** ]  bearing cells such as neutrophils at sites of inflammation.  Ref: 12680872 Clin Exp Allergy, 2003
